# Effects of Lime Powder on the Properties of Portland Cement–Sulphoaluminate Cement Composite System at Low Temperature

**DOI:** 10.3390/ma17153658

**Published:** 2024-07-24

**Authors:** Ge Zhang, Bei Zhang, Yixin Hao, Qianbiao Pang, Lei Tian, Ruyan Ding, Lin Ma, Hui Wang

**Affiliations:** 1 College of Material Science and Technology, Xi’an University of Architecture & Technology, Xi’an 710055, China; 2 Ningbo Key Laboratory of Energy Geostructure, Ningbo 315211, China

**Keywords:** low temperature, lime powder, Portland cement–sulphoaluminate cement

## Abstract

In order to reduce the risk of early freezing damage to cement-based materials in winter construction, lime powder was used to improve the properties of the Portland cement–sulphoaluminate cement (PC–CSA) composite system at low temperatures. In this study, the effects of lime powder dosage on the properties of a PC–CSA blended system with two proportions (PC:CSA = 9:1 and 7:3) at −10 °C were investigated, and the mechanisms of improvement were revealed. The results showed that the compressive strength of the PC–CSA composite system was effectively improved, and the setting time was shortened by the addition of lime powder. Lime powder could effectively act as an early heating source in the PC–CSA composite system, as the maximum temperature of samples exposed to sub-zero temperatures was increased and the time before dropping to 0 °C was prolonged by the addition of lime powder. The extra CH generated by the hydration of lime powder provided an added hydration path for C_4_A_3_$\bar{S}$, which accelerated the formation of AFt at each stage. Frozen water as well as the early frost damage were effectively decreased by lime powder because of the faster consumption of free water at an early stage. The modification of the hydration products also contributed to the denseness of the microstructure.

## 1. Introduction

The difficulty of concrete construction in cold environments was much higher than that in normal temperatures, which limited the development of construction in cold climates [1,2]. This is mainly due to the fact that at low temperatures, the hydration rate of Portland cement (PC) was greatly reduced, and early frost damage was more likely to occur when the temperature was below zero [3]. The results showed that the hydration rate of Portland cement at −10 °C was several orders of magnitude lower than that at normal temperature [4,5]. In addition, after exposure to freezing damage at an early age, the strength of concrete would be reduced by 20~40% [6].

In order to avoid the above problems, it was usually necessary to increase the early temperature by heating water and aggregate, setting up warm sheds, electric blankets, etc., to avoid freezing damage and promote early hydration of cement [2,7,8]. In addition, antifreeze was also commonly used, which also had the effect of preventing freezing damage and promoting cement hydration [7,8]. To ensure effectiveness, both are usually used together. However, these methods had some problems and limitations. For heating methods, it usually had high energy consumption, large carbon emissions, and was not environmentally friendly. Moreover, the commonly used chloride salt antifreeze was easy to cause durability problems in concrete [9,10]. Therefore, safer and more environmentally friendly measures needed to be put forward.

Recently, some scholars have proposed to add sulphoaluminate cement (CSA), which has a faster hydration rate and higher strength development at low temperature, to PC to obtain a PC–CSA composite system with better performance [11,12,13]. Studies had shown that the combination of CSA and PC could have both high early strength and stable late strength, which respectively made up for the shortcomings of low early strength of PC and decreasing strength of CSA at the late stage [12,14]. These excellent properties were related to the special hydration mechanism of the composite system. In the first few minutes of hydration, C_4_A_3_$\bar{S}$, which was the main mineral compound of CSA, was first hydrated to form needle-like ettringite (AFt) crystals, contributing to the formation of early structures (as shown in Equations (1) and (2)) [15]. Subsequently, C_3_S, which was the main mineral compound of PC, was hydrated (as shown in Equation (3)) to form a large amount of C–S–H gel, which was filled in the AFt skeleton to strengthen the microstructure [16]. In addition, the formation of calcium hydroxide (CH) was conducive to the improvement of alkalinity and thus to the stability of AFt [17,18]. Therefore, the composite system had excellent strength properties that cannot be achieved when the two types of cement are used alone.

(1) $$C_{4}A_{3}\bar{S}+2\;C\bar{S}\cdot H_{2}+34H\to C_{3}A\cdot 3\;C\bar{S}\cdot 32H\;+\;2AH_{3}$$

(2) $$C_{4}A_{3}\bar{S}+18H\to C_{3}A\cdot 3\;C\bar{S}\cdot 12H\;+\;2AH_{3}$$

C_3_S + H → C-S-H + CH (3)

However, studies have shown that the appropriate temperature in the first several hours is important for the performance of the PC–CSA composite system at low temperatures [2,19,20]. Li showed that the cracks caused by frost damage in the PC–CSA composite system could be largely reduced by pre-curing at room temperature for 6 h [19]. Zhang’s results showed that at the negative temperature of −5 °C, 2~4 h pre-curing at room temperature was enough for the PC–CSA composite system to make its long-term strength comparable to that of PC curing at 20 °C [2]. During the pre-curing stage, the rapid dissolution of ions and the rapid consumption of free water reduced the freezing point and freezable water of the cement slurry. This not only greatly reduced the risk of freezing damage but also promoted the hydration and early strength of the cement-based material [2]. Therefore, a reasonable heat source at an early stage was necessary for the PC–CSA composite system at low temperatures.

Because of its exothermic characteristics, lime was often used as a heating material in the food field (as shown in Equation (4)) [21,22]. In construction, modified lime can be used as an expansion agent and alkali activator for concrete [23,24,25]. Li [26] first added lime powder as a self-heating material to CSA under a negative temperature of −5 °C. The results showed that the temperature of the system increased from 0 °C to 6 °C in 130 min. In addition to generating heat, lime was also used in the hydration process of CSA. The additional CH formed by lime could be hydrated with C_4_A_3_$\bar{S}$, playing a role in promoting CSA hydration in terms of chemical reactions as well (Equations (5) and (6)) [15]. Therefore, it could be expected that lime powder used as a heating material would improve the performance of the PC–CSA composite system at low temperatures.

CaO + H_2_O → Ca(OH)_2_ + 63.5 kJ/mol (4)

(5) $$C_{4}A_{3}\bar{S}+8C\bar{S}\cdot H_{2}+6\mathrm{CH}+74H\to 3C_{3}A\cdot 3\;C\bar{S}\cdot 32H$$

(6) $$C_{4}A_{3}\bar{S}+2\;C\bar{S}\cdot H_{2}+6\mathrm{CH}+26H\to 3C_{3}A\cdot 3\;C\bar{S}\cdot 12H$$

In this study, the effects of lime powder on the compressive strength, setting time, and freezing performance of the PC–CSA composite system at low temperatures were studied. Five dosages of lime powder (1 wt. %, 2 wt. %, 3 wt. %, 4 wt. %, and 5 wt. %) and two ratios of PC:CSA (9:1 and 7:3) were selected. Additionally, isothermal calorimetry analysis, X-ray diffraction analysis (XRD), scanning electron microscopy (SEM), and back scattering electron analysis (BSE) were used to reveal mechanisms.

## 2. Materials and Methods

### 2.1. Raw Materials

The chemical compositions and physical properties of PC and CSA clinker used in this study are shown in Table 1, Table 2, Table 3 and Table 4 respectively. The CSA was composed of 75% CSA clinker and 25% gypsum. The purity of gypsum was ≥99%. The chemical compositions of lime powder were listed in Table 5, and the purity was ≥95%. The particle distribution of silica sand is shown in Table 6. To obtain the suitable fluidity, polycarboxylate superplasticizer was used.

### 2.2. Mix Proportion

Two PC–CSA composite systems were set up: (1) S10 (the ratio of PC and CSA was 9:1); and (2) S30 (the ratio of PC and CSA was 7:3). In addition, five dosages of lime powder (0 wt. %, 1 wt. %, 2 wt. %, 3 wt. %, 4 wt. %, and 5 wt. %) were added, denoted as C0, C1, C2, C3, C4, and C5. The water-to-cement ratio of binders was 0.26, and the ratio of binder to silica sand was 1:1. The dosage of polycarboxylate superplasticizer was 0.3%.

### 2.3. Specimen Preparation and Curing System

Mortar was prepared to have the test of compressive strength and temperature curve. Considering the actual construction in cold environments, before mixing, solid raw materials were put into the −10 °C chamber, and water was cooled to 5 °C. Powders such as PC, CSA, gypsum, and lime powder were mixed first to make them uniform. Then water and sand were added separately to stir. After preparation, it was poured into the mold and wrapped with polyethylene film. Samples were then put into the negative temperature chamber. Paste was prepared to have the tests of setting time, hydration heat, XRD, SEM, and BSE. The preparation of paste was the same as with mortar, and the only difference was the absence of silica sand.

There were two curing regimes: (1) continuous −10 °C curing for 28 d (−28 d); (2) −10 °C curing for 7 d and then transformed to 20 °C for another 21 d (−7 + 21 d).

### 2.4. Methods

#### 2.4.1. Strength

The compressive strength was measured by an automatic cement strength testing machine, with the rate of 2400 ± 200 N/s. At the age of −1 d, −7 d, −28 d, and −7 + 21 d, mortars with a size of 40 cm × 40 cm × 40 cm were tested. A group of six was tested.

#### 2.4.2. Setting Time

Setting time was tested according to the Chinese standard GB/T 1346-2011 [27], and Vicat apparatus was used.

#### 2.4.3. Temperature Curve

The central temperature of the mortar specimen was measured by the temperature recorder. The initial temperature of the sample was 20 °C, and then it was cooled at −10 °C.

#### 2.4.4. Hydration Heat

Hydration heat of the paste was tested according to the isothermal calorimetry method by using Thermometric TAM Air instrument (TA Instrument Company, New Castle, PA, USA). Ten grams of binder and 5 g of deionized water were used and the test lasted 7 d to 20 °C.

#### 2.4.5. XRD

The hydration products of the composite system were analyzed using XRD. At the specified age, the hardened cement paste was hydrated by isopropyl alcohol. Samples were ground into powder with a size of ≤80 µm. Before the test, 10 wt. % CaF_2_ was blended as the internal standard substance. The XRD test condition was as follows: CuKα target, working voltage 40 kV, working current 40 mA, scanning angle range 2θ = 5~70°, step size 0.02°, and divergence slit 1°. After testing, XRD Rietveld analysis was used to calculate the content of the hydration products.

#### 2.4.6. BSE

The microstructure of the hydration products was observed by back-scattering electron (BSE). Cube samples were embedded in resin after stopping hydration. The resin was polished with 300, 1200, 2000, 4000, 8000, and 10,000 mesh sandpapers in turn. After each polish, the sample needed ultrasonic cleaning to remove excess particles. Binary processing of BSE images was performed to characterize the matrix pore structure more intuitively.

#### 2.4.7. SEM

The morphology of the hydration products was revealed by scanning electron microscopy (SEM). Before the test, 5 mm × 5 mm × 5 mm of hardened cement was taken, and the hydration was stopped with isopropyl alcohol. It was then dried at 40 °C for 48 h.

## 3. Results

### 3.1. Compressive Strength

**Figure 1:**
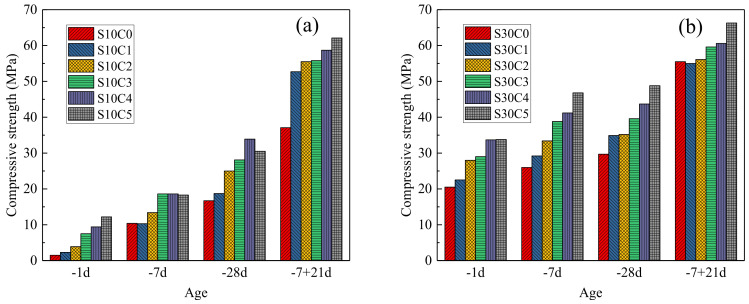
The effects of lime powder dosage on the compressive strength of (**a**) S10 and (**b**) S30.

The effects of lime powder dosage on the compressive strength of S10 and S30 are shown in Figure 1. From Figure 1a, S10 without lime powder had almost no strength at the early age of −1 d at a low temperature of −10 °C. The addition of lime powder significantly increased the early strength. With the increasing dosage of lime powder, the compressive strength continuously increased. When the lime powder dosage was 5 wt. %, the compressive strength of S10C5 was up to 12.2 MPa at −1 d. Under continuously low-temperature curing, the compressive strength of S10 progressively increased. However, the strength was only 16.7 MPa because the low temperature hindered hydration. With lime powder addition, the maximum compressive strength could reach 33.9 MPa for S10C4. When the curing temperature transformed from −10 °C to 20 °C, the compressive strength of S10 rapidly increased. However, because of irreversible freezing damage, the strength was only 37.1 MPa at the late stage of −7 + 21 d. The addition of lime powder obviously enhanced the strength. When the dosage of lime powder was 5 wt. %, the strength was as high as 62.1 MPa. It could be inferred that the addition of lime powder effectively reduced the early freezing damage and promoted the hydration of cement.

When the CSA content increased, as shown in Figure 1b, the early strength was increased to 20.5 MPa at −1 d, indicating that the higher content of CSA significantly promoted the early hydration of the composite system at the low temperature. The addition of lime powder further enhanced the early strength. When the dosage was 5 wt. %, the compressive strength of S30C5 could be as high as 33.8 MPa at −1 d. Under continuously low temperature curing, the compressive strength of S30 was also increased progressively, which was 29.7 MPa at −28 d. But the S30C5 could reach 48.8 MPa due to the presence of lime powder, which accelerated the hydration. When the curing temperature transformed from −10 °C to 20 °C, the compressive strength of S30 also rapidly increased, which was 55.5 MPa at −7 + 21 d. Compared with S10, the acceleration of hydration of S30 with a higher content of CSA at the early stage probably decreased the risk of frosting damage. Therefore, a higher late strength was achieved. The addition of lime powder further increased the compressive strength at this stage. It could be concluded that lime powder could effectively improve the compressive strength of the PC–CSA composite system at various stages. In addition, the promotion effects were more obvious when the CSA content was low.

### 3.2. Setting Time

The effects of lime powder on the setting time of PC–CSA composite systems are shown in Table 7. For S10, the addition of lime powder obviously shortened the initial setting time and final setting time. With the lime powder dosage increasing, the setting time of S10 was continuously shortened. When the dosage of lime powder was 5 wt. %, the initial setting time and final setting time of S10C5 were shortened to 20 min and 29 min, respectively. The shortening of setting time was primarily related to the water consumption of lime powder hydration because 1 mol of CaO consumed 1 mol of water and the hydration was violent [21,22]. Additionally, the hydration of the PC–CSA composite system was probably accelerated by the addition of lime powder. It was mainly due to the release of heat, which accelerated the setting and hardening.

For S30, the increasing CSA content significantly reduced the initial setting time and final setting time compared with S10. With the increase in CSA content, the hydration of the PC–CSA composite system was accelerated. Thus, the setting and the hardening were accelerated. In addition, higher ye’limite led to a greater formation of AFt, of which one molecule contained 32 crystalline waters [28,29]. During the hydration, free water in the cement paste was rapidly consumed. The addition of lime powder further shortened the initial setting time and final setting time. It probably worked the same way as the S10.

### 3.3. Temperature Curve

**Figure 2:**
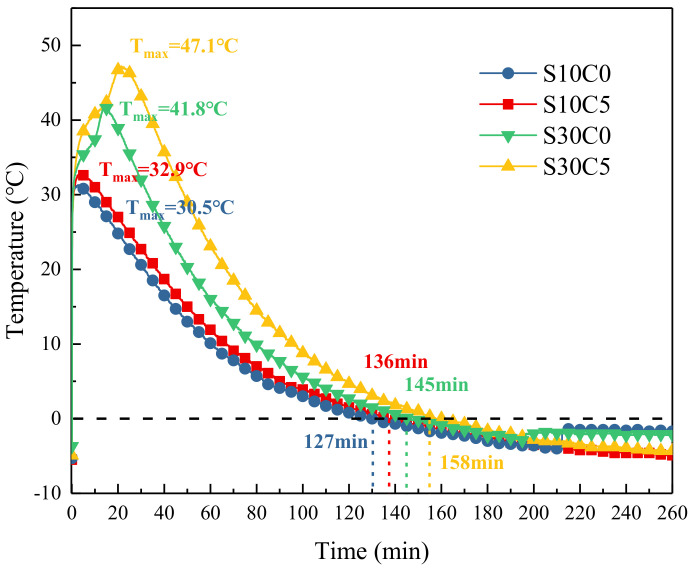
The effects of lime powder addition (5 wt. %) on the temperature curve of samples.

The effects of lime powder on the temperature curves of S10 and S30 are shown in Figure 2. From Figure 2, the temperature of the samples rapidly increased in a few minutes and then slowly reduced to sub-zero temperatures. Compared with samples without lime powder (S10C0 and S30C0), the addition of lime powder obviously enhanced the maximum temperature of the exothermic peak. It was increased from 30.5 °C to 32.9 °C and from 41.8 °C to 47.1 °C for S10 and S30, respectively. It was mainly related to the additional hydration of lime powder and the large hydration heat. In addition, compared with samples with a lower content of CSA (S10), S30 had the higher maximum temperature, and the enhancement of temperature by adding lime powder was more significant. When a PC–CSA composite system contains a higher content of CSA, the early hydration could be accelerated, which would produce more hydration heat. In addition, the chemical reaction between C_4_A_3_$\bar{S}$ (the main mineral composite of CSA) and CH (the hydration product of lime powder) may further accelerate the hydration, as shown in Equations (5) and (6).

Moreover, with the higher heat release, the temperature stayed above zero for longer. It was 127 min, 136 min, 145 min, and 158 min for S10C0, S10C5, S30C0, and S30C5, respectively. Therefore, longer hydration times were provided for samples with lime powder addition and a higher content of CSA.

### 3.4. Hydration Heat

**Figure 3:**
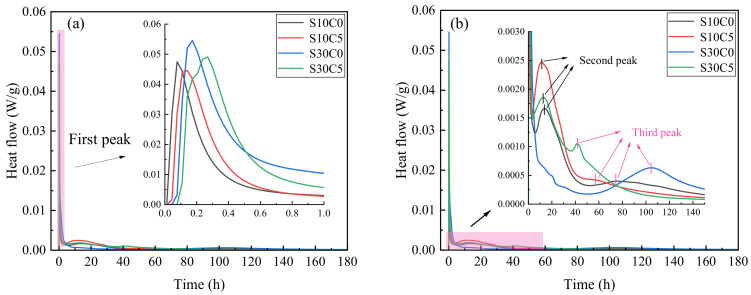
The effects of lime powder addition (5 wt. %) on the hydration heat of samples. (**a**) The first peak; (**b**) The second and third peak.

The effects of lime powder addition (5 wt. %) on the hydration heat of S10 and S30 were shown in Figure 3. From Figure 3, the hydration characteristics of samples were evidently affected by the addition of lime powder and the content of CSA in the PC–CSA composite system.

In the first few minutes (Figure 3a), without lime powder, ions dissolved from cement particles and a small amount of cement hydrated, thus a high exothermic peak appeared [30,31,32]. After adding lime powder, the first exothermic peak was slightly lowered but obviously widened. The lime powder hydrated violently when contacted with water, releasing a large amount of Ca^2+^ and OH^−^ ions. Alizadeh [33] reported that the decrease of Ca^2+^ and OH^−^ ions in pore solution would enhance the local ion concentration around clinker particles, as well as accelerate the subsequent dissolution. The increasing concentration of Ca^2+^ and OH^−^ ions slowed down the dissolution of cement to a certain extent. Thus, the exothermic peak was slightly lowered. Simultaneously, the hydration of lime powder was violent, and the heat was large. Therefore, the exothermic peak was obviously widened. Additionally, compared with S10, higher CSA content led to a higher and wider exothermic peak. It was mainly due to the dissolution of ions, and early hydration was significantly accelerated by the increasing C_4_A_3_$\bar{S}$ in the PC–CSA composite system, which was consistent with the results of the temperature curve [17].

The second exothermic peak was related to the hydration of C_4_A_3_$\bar{S}$ in PC–CSA composite system [2]. For S10, the second exothermic peak was advanced, and the maximum value of the peak was significantly enhanced by the addition of lime powder, indicating that the hydration of C_4_A_3_$\bar{S}$ and the formation of hydration products were promoted. For S30, higher CSA led to the advancement of the second exothermic peak, which was indistinguishable from the first peak. Yang [34] called it “right-angle thickening”. It was mainly due to the fact that C_4_A_3_$\bar{S}$ hydrated rapidly at initial and then directly entered the accelerated period without an induction period.

In the PC–CSA composite system, the third exothermic peak mainly corresponded to the hydration of C_3_S, which was relatively lower and appeared later [2]. For S10 and S30, the third exothermic peak was advanced, and the maximum value of the peak was enhanced by the addition of lime powder, indicating that the hydration of C_3_S was also promoted.

### 3.5. Hydration Products

**Figure 4:**
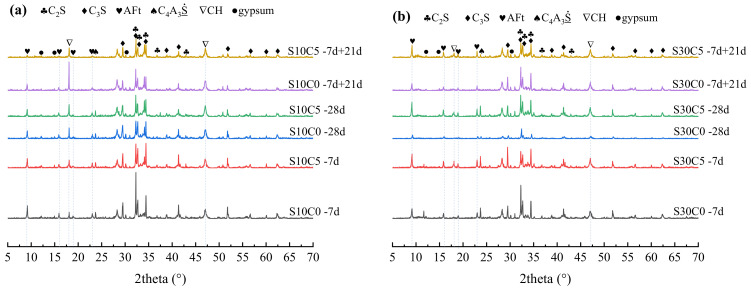
The effects of lime powder addition (5 wt. %) on the XRD patterns of (**a**) S10 and (**b**) S30.

The XRD patterns of samples at different ages are shown in Figure 4. From Figure 4, the types of hydration products of S10C0, S10C5, S30C0, and S30C5 were similar, indicating that the addition of lime powder did not change the hydration product type of the PC–CSA composite system. But the pattern intensity of gypsum and C_4_A_3_$\bar{S}$ was decreased, and simultaneously that of AFt and CH was increased with the growth of age, as well as the lime powder addition. In order to analyze accurately, XRD Rietveld was used to calculate the contents of AFt and CH, as shown in Table 8.

From Table 8, the lime powder addition effectively improved the AFt content in S10 and S30, especially at the early stage. It is not only related to the early release of heat by lime powder, but also to the fact that the hydration of C_4_A_3_$\bar{S}$ was accelerated through other reactions (Equations (5) and (6)). However, with age increasing, AFt transformed into AFm when the gypsum was consumed, and its content was insufficient. Thus, there was a reduction in AFt content for S10 at a late stage. Because S30 possessed a relatively higher gypsum amount, less AFt was converted.

In addition, the content of CH was significantly improved by the addition of lime powder, especially at the early stage. The additional lime powder was hydrated to produce more CH. Moreover, combined with the results of the hydration heat, the hydration of C_3_S was also promoted and contributed to the increase in CH amount.

### 3.6. Pores

**Figure 5:**
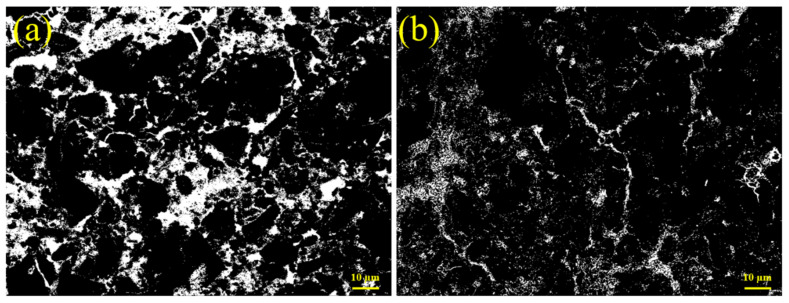
BSE images of S10 cement paste at −7 d. (**a**) Without lime powder; (**b**) with 5 wt. % lime powder.

The grayscale of BSE images could clearly reflect the shape and number of pores. Figure 5 shows the effects of lime powder on the BSE images of S10. After image processing, the brightest parts were pores and cracks, and the dark parts were cement and its hydration products. From Figure 5a, there were a large number of irregular pores in S10C0. The addition of lime powder significantly reduced the size and amount of the pores, as shown in Figure 5b. After adding lime powder, the violent hydration of it would consume a large amount of free water, which reduced the pores caused by the frozen water. In addition, the promotion of hydration by lime powder accelerated the formation of hydration products, which could fill the pores and enhance the density of the microstructure.

**Figure 6:**
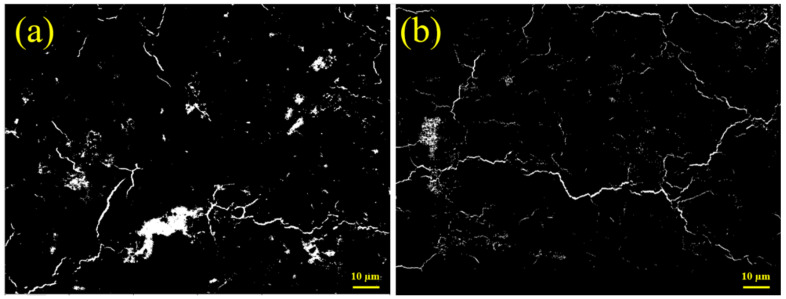
BSE images of S30 cement paste at −7 d. (**a**) Without lime powder; (**b**) with 5 wt. % lime powder.

Figure 6 shows the effects of lime powder on the BSE images of S30. Compared with S10C0, a large number of pores in S30C0 decreased. With a higher content of CSA, free water as well as frozen water were also obviously reduced because of the faster hydration and the large formation of AFt, which was a type of crystal containing a large amount of bound water. Additionally, more hydration products were also conducive to the density of the microstructure. From Figure 6b, the amount and size of pores were further reduced by the addition of lime powder, corresponding to the higher strength.

### 3.7. Morphology and Microstructure

**Figure 7:**
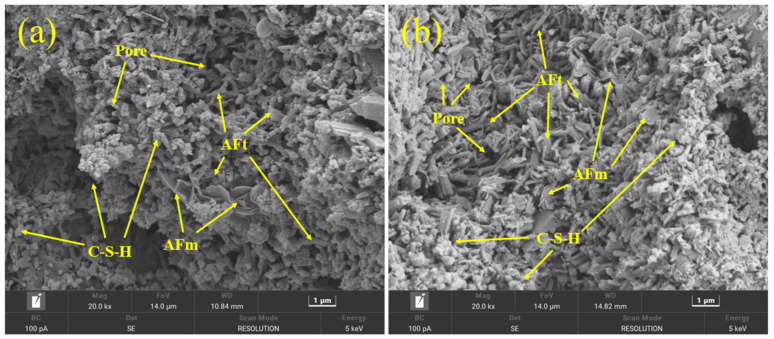
SEM micrographs of S10 cement paste at −7d. (**a**) Without lime powder; (**b**) with 5 wt. % lime powder addition.

The morphology and microstructure of S10 are shown in Figure 7. From Figure 7a, needle-like crystal AFt, amorphous C–S–H gel, uncrystallized AFm, and pores could be observed. Compared with it, adding lime powder increased the amount of AFt, and decreased the amount and size of pores, displaying a denser microstructure (Figure 7b).

**Figure 8:**
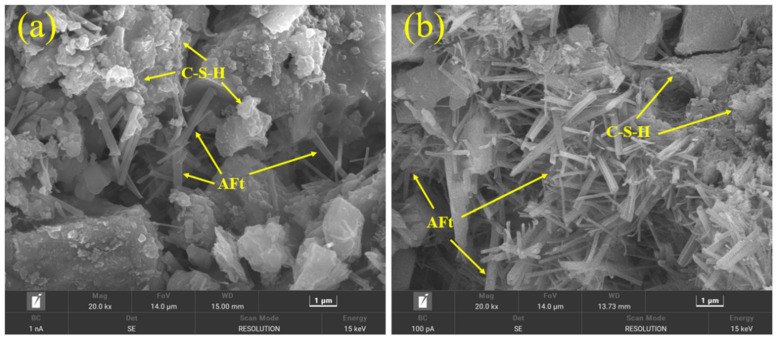
SEM micrographs of S30 cement paste at −7 d. (**a**) Without lime powder; (**b**) with 5 wt. % lime powder addition.

Figure 8 shows the microstructure of S30. Compared with Figure 7, the higher amount of CSA led to a larger crystal size of AFt and a higher polymerization degree of C–S–H gel. In addition, the addition of lime powder further enhanced the crystal amount, resulting in a denser structure (Figure 8b).

**Figure 9:**
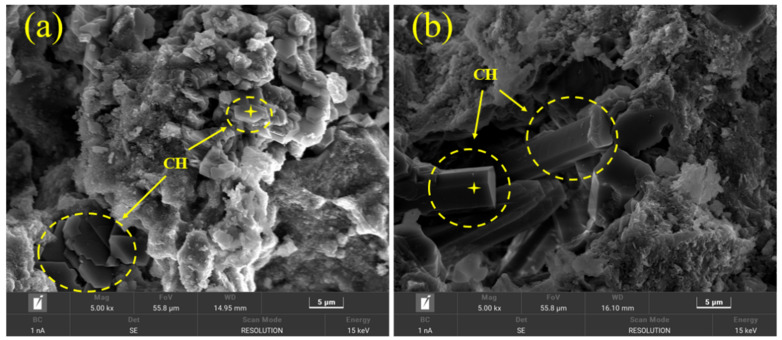
SEM micrographs of CH at −28 d. (**a**) Without lime powder; (**b**) with lime powder addition.

Furthermore, Figure 9 shows the morphology of CH crystal in the PC–CSA composite system without and with lime powder addition. Table 9 and Table 10 are the energy spectrum data of CH in Figure 9a and Figure 9b, respectively. From Figure 9, it could be observed that the morphology of CH crystal changed from hexagonal plates to hexagonal prisms after adding lime powder, as the results of the energy spectrum from Table 9 and Table 10 could demonstrate that they basically belong to one substance. Shen et al. [35] found that the generative habit of CH was in connection with the degree of supersaturation, which could alter the shape of CH. When the supersaturation of CH remained low, CH crystallized through heterogeneous nucleation and two-dimensional surface nucleation, relying on step movement. Thus, CH showed a hexagonal plate shape. With the addition of lime powder, the supersaturation of CH increased significantly. The condition for the three-dimensional nucleation of CH was provided, presenting a prismatic shape. Similar to needle-shaped AFt, the rod-like prismatic shape CH tended to provide higher strength in the microstructure [36].

### 3.8. Mechanisms

**Figure 10:**
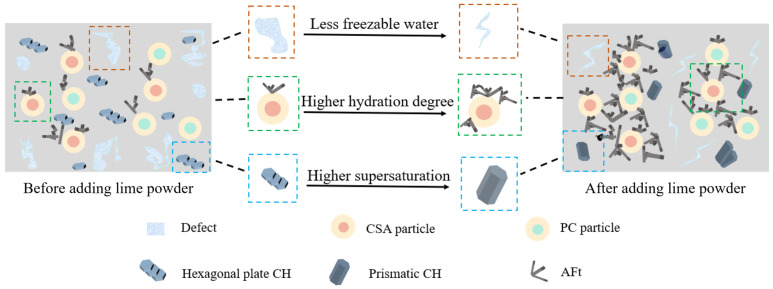
Schematic diagram of the mechanism.

It could be concluded that the performance of the PC–CSA composite system was efficiently enhanced by the addition of lime powder. The enhancement mechanisms could be extracted as follows, and the schematic diagram is shown in Figure 10.

Firstly, the content of freezable water was reduced by the addition of lime powder, resulting in a decrease in the pores. Without lime powder addition, the hydration of cement was hindered by the low temperature, and simultaneously, a large amount of free water existed. Once they were frozen, a large number of pores formed. The addition of lime powder prolonged the time stayed above 0 °C, consumed frozen water by hydration of itself, and accelerated cement hydration. Therefore, the size and number of pores were reduced.

Secondly, the hydration of cement was accelerated, and the amount of the hydration products was significantly enhanced by the addition of lime powder. Before adding lime powder, the development of the early hydration degree of cement was slow at the low temperature. The addition of lime powder greatly increased the number of AFt crystals at an early stage. The cross-overlapping network skeleton structure was beneficial for the development of strength and resistance to freezing damage.

Thirdly, the higher supersaturation of CH was obtained after adding lime powder. In this condition, the morphology of CH was changed from a hexagonal plate shape to a hexagonal prismatic shape. The modification of the microstructure also contributed to the enhancement of strength.

## 4. Conclusions

In this study, the effects of lime powder on the properties of the PC–CSA composite system at −10 °C were investigated. The mechanism of improvement was revealed. The conclusions could be drawn as follows:

(1) The addition of lime powder could effectively improve the compressive strength of the PC–CSA composite system at various stages. The promotion effects were more obvious when the CSA content was low. In addition, the setting time of the PC–CSA composite system was significantly shortened with increasing lime powder dosage.
(2) The lime powder effectively acted as an early heating source in the PC–CSA composite system. The maximum temperature of samples exposed in sub-zero temperatures was increased, and the time before dropping to 0 °C was prolonged by the addition of lime powder. It was not only because of the heat release characteristics of lime itself but also because the hydration heat of the PC–CSA composite system increased.
(3) The hydration of the PC–CSA composite system was not only accelerated by the heat of lime powder but also by the additional CH formed from the hydration of lime powder. Because of the faster consumption of free water, the consumption of frozen water was significantly decreased by the addition of lime powder. Additionally, the pore structures and morphology of hydration products were modified by the lime powder. Therefore, a denser microstructure was obtained, and a higher strength was achieved after adding lime powder.

## Figures and Tables

**Table 1:** Chemical compositions of PC (%).

SiO_2_	Al_2_O_3_	Fe_2_O_3_	CaO	MgO	SO_3_	NaO	f-CaO	Loss	Cl^−^
20.82	4.40	3.27	63.34	2.88	2.42	0.59	0.87	1.54	0.02

**Table 2:** Physical properties of PC.

Specific Surface Area/m^2^/kg	Density g/cm^3^	Setting Time/min		Flexural Strength/MPa			Compressive Strength/MPa		
		Initial	Final	3 d	7 d	28 d	3 d	7 d	28 d
352	3.11	123	188	5.5	7.0	8.8	27.4	36.6	51.2

**Table 3:** Chemical compositions of CSA clinker (%).

SiO_2_	Al_2_O_3_	Fe_2_O_3_	CaO	MgO	SO_3_	TiO_2_
8.2	34.3	2.5	41.8	3.2	8.5	1.0

**Table 4:** Physical properties of CSA clinker.

Specific Surface Area/m^2^/kg	Setting Time/min		Flexural Strength/MPa			Compressive Strength/MPa		
	Initial	Final	6 h	1 d	3 d	6 h	1 d	3 d
464	35	45	6.6	7.4	8.5	29.5	42.3	49.5

**Table 5:** Chemical compositions of lime powder.

CaO	MgO	Fe	P	S	SiO_2_	HCl (Insolubility)
98.2	0.79	0.033	0.0046	0.02	0.17	≤0.03

**Table 6:** Particle distribution of silica sand.

Screen Size (mm)	Cumulative Retained Percentage (%)
4.75	1.1
2.36	6.0
1.18	14.2
0.6	41.6
0.3	86.3
0.15	96.4
<0.15	100

**Table 7:** The effects of lime powder dosage on the setting time (min).

Sample	Initial Setting Time	Final Setting Time
S10C0	75	87
S10C1	47	58
S10C2	38	49
S10C3	28	38
S10C4	26	34
S10C5	20	29
S30C0	19	23
S30C1	18	22
S30C2	15	18
S30C3	13	18
S30C4	12	17
S30C5	9	15

**Table 8:** The contents of AFt and CH in hydration products (%).

Sample	AFt at −7 d	AFt at −28 d	CH at −7 d	CH at −28 d
S10C0	12.76	9.34	2.63	6.76
S10C5	15.62	10.65	9.81	8.81
S30C0	17.32	22.51	2.19	4.39
S30C5	18.27	21.81	7.69	7.64

**Table 9:** Energy spectrum data in Figure 9a.

Chemical Element	wt. %	At %
O	36.33	34.69
Ca	56.42	55.97
C	2.55	5.23
Mg	0.36	0.36
Al	0.51	0.46
Si	3.04	2.67
S	0.79	0.61
Total	100.00	100.00

**Table 10:** Energy spectrum data in Figure 9b.

Chemical Element	wt. %	At %
O	38.58	36.80
Ca	58.23	57.61
C	2.89	5.36
Mg	0.05	0.01
Al	0.05	0.04
Si	0.17	0.14
S	0.06	0.04
Total	100.00	100.00

## Data Availability

The raw data supporting the conclusions of this article will be made available by the authors on request.
